# Application Value of Machine Learning Method in Measuring Gray Matter Volume of AIDS Patients

**DOI:** 10.1155/2022/1210002

**Published:** 2022-06-16

**Authors:** Danhui Fu, Kai Mo, Wenjuan Deng, Yang Zhao, QianLin Ding, Sen Hong, Wei Zhang, Danke Su

**Affiliations:** ^1^Departments of Radiology, Guangxi Medical University Cancer Hospital, Nanning, 530021 Guangxi Zhuang Autonomous Region, China; ^2^Guangxi Key Clinical Specialty (Medical Imaging Department), Dominant Cultivation Discipline of Guangxi Medical University Cancer Hospital (Medical Imaging Department), China; ^3^Department of Neurosurgery, Second Affiliated Hospital of Guangxi Medical University, Nanning, 530021 Guangxi Zhuang Autonomous Region, China; ^4^Department of MR, First Affiliated Hospital of Xinxiang Medical University, Weihui, 453100 Henan Province, China; ^5^Departments of Radiology, Guangzhou Women and Childrens Medical Center, Guangzhou, 510623 Guangdong Province, China; ^6^Departments of Radiology, Liuzhou People's Hospital Affiliated to Guangxi Medical University, Guangxi Zhuang Autonomous Region, China

## Abstract

**Background:**

To investigate the role of gray matter (GM) volume in the identification of HIV-positive patients with HIV-associated neurocognitive impairment (HAND) using a machine learning approach from normal healthy controls.

**Methods:**

Twenty-seven HIV-infected patients and 14 healthy controls were enrolled in our study. Each set of BRAVO images was postprocessed using DPARSF3.1 to coregister all brains on the MNI template, and volume extraction of 90 brain regions was performed using custom-designed code. The machine learning method was performed using PRoNTo2.1.1 toolbox. The differences in brain volume between the HAND and non-HAND groups were analyzed.

**Results:**

GM volume effectively distinguished HIV-positive patients from healthy subjects with an AUC equals to 0.73. The sensitivity, specificity, and accuracy of the established classification were 85.19%, 42.86%, and 70.73%, respectively. GM volume value of the top ten brain regions was related to digit symbols, trail making test, digit span, vocabulary fluency, stroop C time, stroop CW time, CD4, and neuropsychological group.

**Conclusions:**

A machine learning approach facilitates early diagnosis of HAND in HIV patients by MRI-based GM volume measurement.

## 1. Introduction

Acquired immune deficiency syndrome (AIDS) is a disease of immune system caused by human immunodeficiency virus (HIV) infection [1]. Guangxi is the second high prevalence and mortality of HIV infection in China [2]. HIV often involves the central nervous system (CNS) after initial infection [3]. Cognitive and behavioral abnormalities may occur with ongoing CNS inflammation, which are called HIV-associated neurocognitive disorders (HAND) [4]. It has estimated that 15–55% of all HIV-1 cases have HAND [4–6]. Asymptomatic neurocognitive impairment (ANI), mild nerve HIV-associated mild neurocognitive disorder (MND) and HIV-associated dementia (HAD) are different forms of HAND [7]. After highly active antiretroviral therapy (HAART), HAND still exists and affects survival, quality of life, and daily functioning [8]. Therefore, early and accurate diagnosis of HAND is a key factor in improving life quality and prolonging life span. However, at present, the diagnosis of HAND mainly relies on neuropsychological (NP) test, which is subjective and time-consuming [9, 10]. Exploring more reliable and objective methods for diagnosing HAND is essential.

Recent studies have reported changes in the gray matter (GM) volume in HIV-infected patients with HAND [11], which may provide clues for diagnosis of HAND. However, subtle changes of GM volume might be missed. Machine learning method is accurate and objective for diagnosing HAND using high-resolution anatomical data provided by MR imaging.

As a technique for identifying patterns that can be applied to medical images, machining learning allows computers to automatically learn the rules from the data and use them to predict unknown data [12]. The machine learning method presents the weight of each brain region difference in the two groups and finds the brain region with the most significance. Support vector machine (SVM) is a pattern recognition method based on statistical learning theory. The main use of SVM is solving small sample, high-dimensional, and nonlinear problems. Compared with traditional statistical analysis methods, SVM has better generalization ability. Therefore, machine learning may be a noninvasive and objective method of early warning of HAND and evaluation of efficacy. The purpose of this study is to explore the application value of machine learning method in measuring GM volume of AIDS patients.

## 2. Methods

This study was approved by Affiliated Tumor Hospital of Guangxi Medical University and the Fourth People's Hospital of Nanning. All participants signed informed consent.

### 2.1. Subjects

Twenty-seven HIV-infected patients (14 males, 13 females; mean age: 42.48 ± 13.03 years; age range: 22-63years) and 14 healthy controls (8 males, 6 females; mean age: 39.0 ± 13.02 years; age range: 22-63years) were enrolled in our study. The HIV-infected patients first diagnosed at the Fourth People's Hospital of Nanning from Sep. 2017 to Jan. 2019 were enrolled in our study. Urban area, age, and gender of controls were highly matched with patients. The inclusion criteria for patients included patients who can move freely and did not perform HAART. The exclusion criteria for all subjects included any drug abuse history and any obvious brain structural abnormalities or lesions, such as stroke or tumors.

### 2.2. MRI Acquisition

All the MRI scans were acquired on GE Discovery MR 750w 3.0T (Avanto, Siemens, Erlangen, Germany). Two triangular cylindrical sponges were used for reducing involuntary movement of head, and earplugs were used for eliminating noises. Axial T1WI and axial T2WI scannings were performed. Axial T1WI-flair (TR 1750 ms, TE 24 ms) and axial T2WI (TR 3500 ms, TE 102 ms) were obtained. Sag3D T1WI-BRAVO were obtained with the following parameters: TR = 7 ms; TE = 3 ms; in-plane matrix = 256 × 256; FOV = 240 × 240 mm; NEX = 1; flip angle = 12°; slice thickness = 1 mm; slice gap = 0 mm; voxel size = 1 × 1 × 1 mm^3^.

### 2.3. Image Preprocessing

DICOM data of subjects were obtained from Siemens company'sADW42 workstation for image preprocessing. The image was preprocessing using statistical parametric mapping software (SPM12) and data processing assistant for resting-state fMRI (DPARSF3.1) in matlab2013b. Then, the specific preprocessing steps were as follows: firstly, we selected TI DICOM to NIFTI to convert the DICOM format to NIFTI format, then readjusted the direction of the T1 image (Reorient T1), and then, performed new segmentation and registration with the DARTEL template (New segment + DARTEL); finally, smoothing processing was performed to reduce deformation and noise caused by radiation transformation. Therefore, sign-noise ratio was evaluated.

### 2.4. Calculation and Presentation of Results

PRoNTo machine learning toolkit (PRoNTo 2.1.1) was used for statistical analysis. Specific steps were as follows: load data: input data according to AIDS group and control group, and the age was added in the modalities as covariate to remove the age influences on brain function. Prepare feature set: set voxel-based morphological measurement method. Select model and run model: selected classification and cross-validation, leaved one subject per group out, and choosing normalize samples and regress out covariates subject level for data operation. Computer weights: get the PRT diagram and add the AAL template. Display results: the PRT map generated in the previous step is imported to obtain the receiver operating characteristic (ROC) curve, the total accuracy value, and the confusion matrix. The sensitivity, specificity, positive predictive value (PPV), and negative predictive value (NPV) of the VBM index are calculated by the confusion matrix diagram. Last, display weights: the final generated PRT map is also imported, and the weights per region are selected. The weights are ranked in order, and the top ten brain regions are selected as the result.

### 2.5. Statistical Analysis

HIV-infected group was divided into HAND group (ANI group, MND group and HAD group) and non-HAND group with reference to NP test. The difference of structural image gray matter volume between two groups was analyzed by independent-sample *T* test using SPSS19.0. The diagnostic value of GM volume value in HAND was investigated. The GM volume of top ten brain regions with biggest differences were used to explore the relationship between GM volume and the clinical hematological index, clinical scale [2]. Spearman rank correlation analysis of ordinal data and Pearson linear correlation analysis of count data were performed. The correlation coefficient *R* greater than 0.6 was highly correlated, 0.4-0.6 was moderately correlated, and less than 0.4 was mildly correlated. *P* < 0.05 was considered statistically significant.

## 3. Result

### 3.1. The Top Ten Brain Regions with Biggest GM Volume Differences between AIDS and Control by SVM

**Figure 1:**
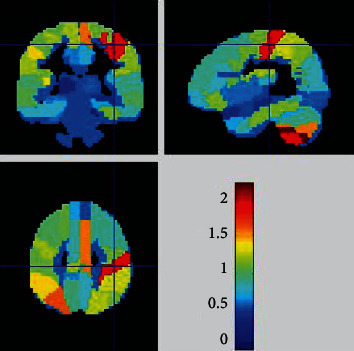
A weight map of the brain regions contributing to the evaluation of the difference between the AIDS group and the control group by gray matter volume in machine learning. The larger the weight, the closer the color of the brain area is to red, and the smaller the weight, the closer the color of the brain area is to blue.

The top ten brain regions that contributed the most to the difference in gray matter outcomes between AIDS patients and control groups in linear support vector machine classification were the right postcentral gyrus, left superior parietal gyrus, right paracentral lobule, right supplementary motor area, left lateral inferior parietal angular gyrus, left lateral temporal gyrus, right inferior parietal angular gyrus, right superior parietal gyrus, right central lid sulcus, and left superior marginal gyrus. The corresponding weight values and region of interest (ROI) values were presented in Table 1 and Figure 1.

### 3.2. Evaluation of the Classification Effect of SVM on GM Volume

**Figure 2:**
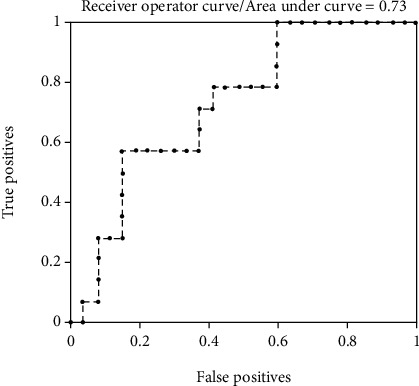
The ROC curve graph of gray matter volume in evaluating the difference between the AIDS group and the control group in machine learning. The horizontal axis represents the negative samples (true positives) predicted by the model as positive, and the vertical axis represents the positive samples predicted by the model as positive (false positives), the area under the ROC curve = 0.73, which means that the gray matter volume has a certain accuracy in evaluating the difference between the HIV-positive group and the control group.

The AUC value, accuracy rate, sensitivity, specificity, positive predictive value, and negative predictive value were 0.73, 70.73%, 85.19%, 42.86%, 74.19%, and 60.00%, respectively. Specific results were shown in Figure 2.

### 3.3. Evaluation of GM Volume Index on HAND Diagnosis

The GM volume values of each brain region in the HAND group and the non-HAND group were shown in Table 2. The GM volume of HAND group was decreased than non-HAND group. We found that in the AAL template, GM volume differences between two groups in the brain regions of nos. 42, 49, 52, 54,73, 74, 75, and 76 were not statistically significant, while 82 remaining brain regions were significant statistically, among which there were 45 brain regions with *P* value < 0.005, as shown in Table 2.

### 3.4. Correlation Analysis

Correlation analysis of GM volume of the top ten brain regions and clinical index, clinical scale, and NP group in AIDS and control group was performed. We found that GM volume value of the top ten brain regions was related to digit symbols, trail making test, digit span, vocabulary fluency, stroop C time, stroop CW time, CD4, and NP group. The degree of correlation was moderate or highly positive or negative correlation except the right parietal superior gyrus. Specific results were as follows: The GM volume of the right postcentral gyrus was highly negatively correlated with stroop C time. The GM volume of left superior parietal gyrus was highly related with stroop C time. The GM volume of right paracentral gyrus and left parietal margin angular gyrus were highly negatively correlated with stroop C time, stroop CW time, and NP group. The GM volume of right supplementary motor area was highly positively correlated with the digit symbol and was highly negatively correlated with stroop C time, stroop CW time, and NP group. The GM volume of the left heschl gyrus was highly negatively correlated with stroop CW time. The GM volume of the right parietal margin angular gyrus was highly positively correlated with the CD4 and highly negatively correlated with stroop C time, stroop CW time, and NP group. The GM volume of right superior parietal gyrus was highly positively correlated with digit symbols, and highly negatively correlated with stroop C time and stroop CW time. The GM volume of right rolandic operculum was highly positively correlated with digit symbols and vocabulary fluency and highly negatively correlated with stroop C time, stroop CW time, and NP group. The GM volume of left supramarginal gyrus was slightly positively correlated with CD4/CD8, and the correlation with the other indicators was moderately positive or negative. All results are shown in Table 3.

## 4. Discussion

In this study, we evaluated the difference of GM volume between AIDS group and control group and explored the correlation of GM volume and clinical index and NP group. The results showed that the GM volume of HAND group was decreased than non-HAND group, and the top ten brain regions with biggest GM volume differences between AIDS and control were the right postcentral gyrus, left superior parietal gyrus, right paracentral gyrus, right supplementary motor area, left parietal margin angular gyrus, left heschl gyrus, right parietal margin angular gyrus, right superior parietal gyrus, right rolandic operculum, and left supramarginal gyrus. The top ten brain regions were concentrated in the bilateral frontal lobe, bilateral parietal lobe, and left temporal lobe. GM volume values of the top ten brain regions were highly correlated with clinical index and NP group. The area under the ROC curve of GM volume in machine learning to evaluate differences between AIDS group and control group is 0.73.

At present, the diagnosis of HAND mainly depends on NP test, which is subjective and lacks of accuracy. Additionally, it is difficult to find subtle changes of GM in human diagnosis. Therefore, it is easy to miss diagnosis of HAND. The volume value of GM obtained by machine learning method can directly reflect the extent of damage to the regions and corresponding functions of the impaired cognitive dysfunction, which plays an important role in improving the clinical antiviral treatment program for patients, increasing the intervention measures of neurocognitive impairment, and reducing the occurrence and development of HAND. Correlation analysis of GM values in areas of the brain with positive manifestations may provide an objective way to evaluate the efficacy of patients. The area under the ROC curve of GM volume in machine learning to evaluate differences between AIDS group and control group is 0.73, which indicated that change of GM volume may help early diagnosis of HAND. Recent studies reported that prefrontal GM atrophy in HIV patients is associated with prolonged disease duration, and motor dysfunction is associated with basal ganglia gray matter atrophy [13]. Therefore, application of machining learning method in measuring GM volume is of great importance.

There are many studies on gray matter volume in HIV patients. Studies of early AIDS have pointed out that cognitive impairment in HAND is associated with early subcortical and cerebral frontal lobe damage [6]. Becker et al. [14] found that HIV-related reductions in GM volume include the posterior and inferior temporal lobe, parietal lobe, and cerebellum. Pluta et al. [15] found that the volumes of the caudate nucleus, hippocampus, insular lobe, and subfrontal gyrus and GM were smaller in seropositive subjects compared with that in healthy controls. These patients behaved worse in cognitive fluency tasks. Küper et al. [13] proposed that compared with the control group, the HIV-positive patients with cognitive impairment showed reduced anterior cingulate gyrus and temporal cortex GM and the white matter of the midbrain. Our study showed the ten brain regions mainly concentrated in the bilateral frontal lobe, bilateral parietal lobe, and left temporal lobe, which was consistent with previous studies [13–16]. However, the change of GM volume in right rolandic operculum of HIV-infected patients has not been reported. Bilateral rolandic operculum damage leads to language suppression [17]. This requires further research to confirm.

There are several limitations of this study that need to be considered. First of all, small participant cohort may have an effect on the power of the statistical analysis in our study. However, SVM is suitable for small sample, which may make our results more reliable. Establishing a data base which contains all related information is a good way to analyze and predict HAND. Second, our subjects only include adults with wide age range. Childhood AIDS were not included in our study. Thus, further study should include paediatric cohorts. The last inevitable limitation was that some HIV-infected patients are not appropriate for MR imaging, and we cannot obtain more comprehensive data.

## 5. Conclusions

Machine learning is of significance in the classification of GM volume measurement in patients with AIDS based on MRI, contributing to early diagnosis of HAND.

## Figures and Tables

**Table 1:** Top ten brain regions with the largest difference in gray matter volume between AIDS patients and controls in linear support vector machine classification.

Area	Region of interest (ROI)	ROI weight (%)
Right postcentral gyrus	8056	1.84
Left superior parietal gyrus	4592	1.59
Right paracentral lobule	1600	1.54
Right supplementary motor area	5136	1.52
Left inferior parietal gyrus	5408	1.41
Left heschl gyrus	576	1.35
Right inferior parietal gyrus	3336	1.27
Right superior parietal gyrus	4592	1.24
Right rolandic operculum	3144	1.21
Left supramarginal gyrus	2808	1.20

**Table 2:** Evaluation value of gray matter volume index in HAND group and non-HAND group.

Brain area number	Non-HAND group (mean ± SD)	HAND group (mean ± SD)	*T* value	*P* value
h1	0.34 ± 0.046	0.29 ± 0.029	3.064	0.005
h2	0.32 ± 0.031	0.28 ± 0.035	3.229	0.003
h3	0.31 ± 0.034	0.26 ± 0.036	3.355	0.003
h4	0.33 ± 0.040	0.28 ± 0.034	3.029	0.006
h5	0.41 ± 0.049	0.35 ± 0.044	3.367	0.002
h6	0.41 ± 0.049	0.35 ± 0.042	3.328	0.003
h7	0.36 ± 0.046	0.31 ± 0.035	3.079	0.005
h8	0.38 ± 0.044	0.33 ± 0.036	3.592	0.001
h9	0.40 ± 0.048	0.35 ± 0.045	2.981	0.006
h10	0.42 ± 0.064	0.36 ± 0.049	2.612	0.015
h11	0.37 ± 0.054	0.29 ± 0.038	4.317	0.000
h12	0.37 ± 0.038	0.30 ± 0.042	3.904	0.001
h13	0.34 ± 0.054	0.28 ± 0.036	3.546	0.002
h14	0.32 ± 0.056	0.26 ± 0.034	3.597	0.001
h15	0.36 ± 0.045	0.32 ± 0.039	2.836	0.009
h16	0.36 ± 0.052	0.31 ± 0.041	2.647	0.014
h17	0.42 ± 0.060	0.36 ± 0.041	3.254	0.003
h18	0.43 ± 0.049	0.36 ± 0.046	3.920	0.001
h19	0.35 ± 0.031	0.31 ± 0.031	2.927	0.007
h20	0.36 ± 0.031	0.31 ± 0.028	4.059	0.000
h21	0.51 ± 0.050	0.44 ± 0.045	3.497	0.002
h22	0.49 ± 0.052	0.43 ± 0.051	2.873	0.008
h23	0.35 ± 0.036	0.31 ± 0.037	2.911	0.007
h24	0.36 ± 0.038	0.31 ± 0.041	3.162	0.004
h25	0.44 ± 0.055	0.37 ± 0.044	3.266	0.003
h26	0.45 ± 0.054	0.38 ± 0.053	3.172	0.004
h27	0.49 ± 0.051	0.41 ± 0.048	3.928	0.001
h28	0.49 ± 0.059	0.41 ± 0.047	4.112	0.000
h29	0.48 ± 0.034	0.43 ± 0.040	3.173	0.004
h30	0.49 ± 0.038	0.44 ± 0.045	2.958	0.007
h31	0.44 ± 0.050	0.37 ± 0.046	3.702	0.001
h32	0.42 ± 0.059	0.35 ± 0.044	3.292	0.003
h33	0.44 ± 0.042	0.38 ± 0.041	3.477	0.002
h34	0.46 ± 0.044	0.40 ± 0.041	3.988	0.001
h35	0.37 ± 0.040	0.31 ± 0.045	3.198	0.004
h36	0.32 ± 0.036	0.28 ± 0.038	2.989	0.006
h37	0.47 ± 0.058	0.42 ± 0.041	2.470	0.021
h38	0.45 ± 0.044	0.41 ± 0.042	2.202	0.037
h39	0.48 ± 0.054	0.43 ± 0.035	3.002	0.006
h40	0.50 ± 0.056	0.45 ± 0.037	2.811	0.009
h41	0.58 ± 0.048	0.54 ± 0.042	2.468	0.021
h42	0.55 ± 0.046	0.52 ± 0.035	1.909	0.068
h43	0.42 ± 0.036	0.38 ± 0.053	2.110	0.045
h44	0.42 ± 0.030	0.38 ± 0.055	2.154	0.041
h45	0.37 ± 0.036	0.33 ± 0.039	2.574	0.016
h46	0.37 ± 0.035	0.33 ± 0.041	2.367	0.026
h47	0.43 ± 0.046	0.39 ± 0.047	2.325	0.028
h48	0.43 ± 0.041	0.39 ± 0.049	2.257	0.033
h49	0.32 ± 0.036	0.30 ± 0.037	1.229	0.230
h50	0.33 ± 0.040	0.30 ± 0.031	2.352	0.027
h51	0.40 ± 0.047	0.36 ± 0.037	2.619	0.015
h52	0.40 ± 0.049	0.36 ± 0.046	1.988	0.058
h53	0.41 ± 0.046	0.36 ± 0.054	2.539	0.018
h54	0.37 ± 0.032	0.35 ± 0.056	1.097	0.283
h55	0.54 ± 0.057	0.49 ± 0.039	2.933	0.007
h56	0.55 ± 0.060	0.49 ± 0.052	2.910	0.007
h57	0.31 ± 0.032	0.26 ± 0.036	3.436	0.002
h58	0.31 ± 0.037	0.26 ± 0.035	3.164	0.004
h59	0.32 ± 0.033	0.27 ± 0.032	3.765	0.001
h60	0.28 ± 0.028	0.24 ± 0.027	3.425	0.002
h61	0.39 ± 0.039	0.33 ± 0.041	3.763	0.001
h62	0.42 ± 0.038	0.36 ± 0.038	4.023	0.000
h63	0.41 ± 0.073	0.33 ± 0.053	3.029	0.006
h64	0.42 ± 0.061	0.34 ± 0.043	3.986	0.001
h65	0.43 ± 0.053	0.35 ± 0.044	3.904	0.001
h66	0.40 ± 0.046	0.36 ± 0.039	2.349	0.027
h67	0.38 ± 0.038	0.34 ± 0.037	2.842	0.009
h68	0.41 ± 0.039	0.36 ± 0.038	3.011	0.006
h69	0.27 ± 0.022	0.24 ± 0.024	2.714	0.012
h70	0.29 ± 0.024	0.25 ± 0.029	3.444	0.002
h71	0.41 ± 0.067	0.36 ± 0.041	2.421	0.023
h72	0.41 ± 0.063	0.36 ± 0.037	2.325	0.028
h73	0.51 ± 0.054	0.48 ± 0.044	1.664	0.109
h74	0.51 ± 0.048	0.48 ± 0.039	1.788	0.086
h75	0.21 ± 0.020	0.21 ± 0.025	.320	0.751
h76	0.19 ± 0.017	0.18 ± 0.020	.397	0.695
h77	0.26 ± 0.037	0.22 ± 0.045	2.250	0.034
h78	0.29 ± 0.035	0.25 ± 0.040	2.681	0.013
h79	0.43 ± 0.075	0.36 ± 0.051	3.094	0.005
h80	0.43 ± 0.054	0.34 ± 0.052	4.311	0.000
h81	0.39 ± 0.072	0.33 ± 0.050	2.740	0.011
h82	0.40 ± 0.060	0.34 ± 0.040	3.101	0.005
h83	0.36 ± 0.040	0.31 ± 0.037	3.519	0.002
h84	0.37 ± 0.036	0.32 ± 0.032	3.563	0.002
h85	0.42 ± 0.057	0.36 ± 0.047	2.882	0.008
h86	0.43 ± 0.058	0.37 ± 0.048	2.783	0.010
h87	0.46 ± 0.065	0.39 ± 0.051	3.112	0.005
h88	0.42 ± 0.049	0.36 ± 0.039	3.729	0.001
h89	0.47 ± 0.059	0.39 ± 0.050	3.328	0.003
h90	0.47 ± 0.057	0.40 ± 0.051	3.193	0.004

**Table 3:** Correlation analysis of gray matter volume in the top ten brain regions with clinical index, clinical scale, and NP group in AIDS patients.

	Right postcentral gyrus		Left superior parietal gyrus		Right paracentral lobule		Right supplementary motor area		Left inferior parietal gyrus		Left heschl gyrus		Right inferior parietal gyrus		Right superior parietal gyrus		Right rolandic operculum		Left supramarginal gyrus	
	*R*	*P*	*R*	*P*	*R*	*P*	*R*	*P*	*R*	*P*	*R*	*P*	*R*	*P*	*R*	*P*	*R*	*P*	*R*	*P*
Digit symbol test	0.578	0.002	0.565	0.002	0.552	0.003	0.676	0	0.595	0.001	0.499	0.008	0.524	0.005	0.628	0	0.637	0	0.515	0.006
Trail making test	-0.448	0.019	-0.463	0.015	-0.287	0.147	-0.484	0.011	-0.566	0.002	-0.338	0.04	-0.404	0.036	-0.593	0.001	-0.461	0.016	-0.428	0.026
Digit span test	0.446	0.02	0.478	0.012	0.238	0.232	0.416	0.031	0.432	0.024	0.445	0.085	0.312	0.113	0.427	0.026	0.431	0.025	0.301	0.126
Vocabulary fiber	0.595	0.001	0.448	0.019	0.442	0.021	0.538	0.004	0.483	0.011	-0.564	0.02	0.302	0.126	0.475	0.012	0.618	0.001	0.449	0.019
Stroop C	-0.678	0	-0.618	0.001	-0.677	0	-0.693	0	-0.746	0	-0.636	0	-0.702	0	-0.745	0	-0.667	0	-0.596	0.001
Stroop CW	-0.573	0.002	-0.591	0.001	-0.69	0	-0.636	0	-0.631	0	0.519	0.006	-0.688	0	-0.62	0.001	-0.69	0	-0.484	0.011
CD4	0.461	0.016	0.506	0.007	0.434	0.024	0.468	0.014	0.567	0.002	0.453	0.018	0.661	0	0.562	0.002	0.437	0.023	0.506	0.007
CD4/CD8	0.355	0.069	0.437	0.023	0.33	0.093	0.332	0.091	0.478	0.012	0.285	0.149	0.503	0.007	0.418	0.03	0.341	0.081	0.382	0.049
Wisconsin card	0.276	0.163	0.112	0.579	0.435	0.024	0.253	0.203	0.222	0.266	-0.554	0.003	0.219	0.002	0.374	0.055	0.581	0.001	0.479	0.011
NP group	-0.576	0.002	-0.587	0.001	-0.725	0	-0.687	0	-0.623	0.001			-0.746	0.272	0.166	0.407	0.355	0.069	0.161	0.422
														0.027	-0.163	0.417	-0.316	0.109	-0.336	0.087
														0	-0.579	0.002	-0.677	0	-0.495	0.009

## Data Availability

The authors confirm that the data supporting the findings of this study are available within the article.
